# Beyond Pain Relief: Motor Effects of Spinal Cord Stimulation in Chronic Incomplete Spinal Cord Injury During Routine Outpatient Rehabilitation—A Case Report

**DOI:** 10.3390/jcm15176899

**Published:** 2026-09-06

**Authors:** Maximilian Wankner, Rene Marquez-Franco, Simon Stork, Paul Kirchner, Petra Heiden, Veerle Visser-Vandewalle, Pablo Andrade

**Affiliations:** 1 Department of Stereotactic and Functional Neurosurgery, Faculty of Medicine and University Hospital Cologne, University of Cologne, 50937 Cologne, Germany; 2 Department of Neurosurgery, Faculty of Medicine and University Hospital Cologne, University of Cologne, 50937 Cologne, Germany; 3 Therapie am Hafen, 20459 Hamburg, Germany

**Keywords:** spinal cord injury, spinal cord stimulation, conus medullaris, neuromodulation, rehabilitation, neuropathic pain, dynamometry

## Abstract

**Background**: Spinal cord injury (SCI) is associated with persistent motor impairment, neuropathic pain, and reduced quality of life. While spinal cord stimulation (SCS) is an established therapy for neuropathic pain, motor effects have largely been demonstrated in experimental settings with activity-based training protocols. Whether such effects can be achieved within routine clinical care remains unclear. **Case Presentation**: We report on motor effects after the clinical implementation of percutaneous SCS using a commercially available system in a patient with chronic, posttraumatic, incomplete thoracic SCI. Following a standard inpatient trial and implantation of two percutaneous leads for neuropathic pain, all subsequent management, programming, and rehabilitation were conducted in an outpatient setting. Task-specific stimulation programs were iteratively developed to support functional activities during routine rehabilitation. Lower-extremity muscle strength was assessed using standardized handheld dynamometry at baseline and at monthly intervals over six months under stimulation-ON and stimulation-OFF conditions. **Results**: Serial dynamometry showed early stimulation-associated increases in force generation, with higher values under stimulation-ON than stimulation-OFF conditions. Over time, absolute strength values under OFF conditions also increased across individual muscle measurements, while stimulation-associated ON–OFF differences became less pronounced during intermediate follow-up but remained evident at month six. These observations included both acute stimulation-associated differences in force generation and longitudinal improvements in OFF-state motor performance. Stimulation was well tolerated during daily use and successfully integrated into outpatient rehabilitation. Functional outcomes showed concordant improvements in ambulatory performance and patient-reported domains. No stimulation-related adverse effects were observed during follow-up. **Conclusions**: This case illustrates how clinically indicated percutaneous SCS can be combined with motor-oriented programming within routine outpatient rehabilitation. The observed stimulation-associated motor effects and longitudinal changes in stimulation-OFF strength are limited to this individual patient and cannot establish efficacy or causality. Prospective controlled studies are needed to determine the reproducibility and clinical relevance of these observations in chronic incomplete SCI.

## 1. Introduction

Spinal cord injury (SCI) is frequently associated with persistent motor deficits, neuropathic pain, and reduced quality of life [1,2]. In the chronic phase, therapeutic options aimed at meaningful neurological recovery remain limited, and treatment strategies are typically focused on symptom control and functional compensation rather than restoration of motor function [3]. While rehabilitation can stabilize or modestly improve performance, its ability to directly modulate spinal network excitability is limited [3].

Spinal cord stimulation (SCS) is an established therapy for chronic pain in diverse pathologies [4]. Beyond its analgesic effects, experimental and recent clinical studies suggest that electrical stimulation of spinal networks can increase excitability of residual neural circuits and facilitate voluntary motor output, particularly when combined with task-specific training [5,6,7]. However, these approaches have predominantly been investigated in highly controlled research settings, often involving specialized stimulation systems and intensive inpatient rehabilitation protocols [5,8]. In contrast, evidence regarding the integration of clinically available, percutaneous SCS systems, implanted for established pain indications, into routine outpatient rehabilitation in patients with chronic SCI is limited. In particular, it remains unclear whether motor-relevant effects can be observed and objectively captured under real-world conditions without specialized infrastructure.

Here, we describe the implementation of percutaneous SCS using a commercially available system for chronic pain in a patient with chronic incomplete SCI. We focus on the integration of neuromodulation into routine outpatient care, including task-specific stimulation programming during physiotherapy and longitudinal objective assessment of lower-extremity strength. This report aims to characterize both immediate stimulation-associated effects and longitudinal changes in motor performance relative to baseline under real-world conditions.

## 2. Case Presentation

A 62-year-old man with a history of traumatic SCI was evaluated for chronic neuropathic pain and persistent lower-extremity motor impairment. The patient had sustained an incomplete thoracic SCI following a fall and underwent decompressive laminectomy at T8–T10 five years before evaluation. At the time of the initial injury, neurological assessment classified the injury as American Spinal Injury Association (ASIA) Impairment Scale grade C (AIS C) [9]. At the pre-implantation assessment five years later, neurological status was classified as AIS D with a neurological level of injury at L2 and a Lower Extremity Motor Score (LEMS) of 36/50; light-touch and pinprick sensation were intact throughout the ISNCSCI examination.

At presentation for SCS evaluation, the patient exhibited chronic paraparesis with preserved but limited voluntary motor function below the level of injury. Functionally, he was primarily wheelchair-dependent and able to ambulate only a few steps with a walking frame and assistance. Previous inpatient and outpatient rehabilitation had resulted in a stable functional plateau, with no further meaningful improvement beyond one year post-injury.

The patient reported chronic neuropathic and musculoskeletal pain, characterized by persistent burning pain in both lower extremities and the gluteal region, accompanied by moderate spasticity. Pain intensity ranged from 5 to 8 out of 10 on the Numeric Rating Scale (NRS). Previous conservative treatment included pregabalin at a dose of 150 mg/day for neuropathic pain, which was discontinued because it did not provide meaningful pain relief, and baclofen at a dose of 5 mg three times daily for spasticity, which was discontinued because of treatment-related fatigue. Metamizole was used intermittently for pain, with limited subjective benefit. At the time of SCS evaluation, the patient was not receiving regular pharmacological treatment specifically for neuropathic pain or spasticity. His regular medication was limited to antihypertensive therapy. Therapeutic alternatives consisted primarily of continued conservative management, including pharmacological treatment as tolerated and ongoing physiotherapy. Given the persistence of functionally limiting neuropathic pain despite previously attempted conservative treatment, SCS was considered as an established treatment option for neuropathic pain.

## 3. Device and Implantation

After careful evaluation of the pain condition, the patient underwent implantation of an SCS system for the treatment of chronic neuropathic pain in September 2025. Preoperative thoracolumbar magnetic resonance imaging (MRI) confirmed that the conus medullaris was located at the T11–L1 vertebral levels. Two percutaneous epidural leads (Evoke CAP12™, Saluda Medical, Macquarie Park, Australia), each containing 12 contacts, were implanted under fluoroscopic guidance with the patient under light sedation. Intraoperative paresthesia mapping was performed to optimize lead placement, with final positioning at the T11–L1 region based on complete coverage of the patient’s painful areas. The leads were placed in parallel parasagittal positions to maximize bilateral paresthesia coverage and pain control. Intraoperative testing confirmed appropriate stimulation-induced paresthesias. The anatomical localization of the conus on preoperative MRI additionally allowed stimulation to be delivered in a region where segmentally organized motor responses could be assessed. Following implantation, the leads were connected to externalized extensions and an external pulse generator for a five-day trial period. During the trial period, one lead required revision because of migration, after which adequate positioning and stimulation coverage were restored. Following a favorable clinical response, defined as pain reduction greater than 50%, a permanent implantable pulse generator (Evoke^®^ Closed Loop Stimulator, Saluda Medical) was implanted in a subcutaneous gluteal pocket. No perioperative neurological or infectious complications occurred.

## 4. Stimulation Programming and Clinical Course

Following implantation, individualized programming was performed as part of routine clinical care. Initial programming focused on pain control, patient comfort, and optimization of paresthesia coverage. The implanted system allowed the configuration of four separate stimulation programs, which were iteratively adjusted over the postoperative course according to clinical needs and patient-reported effects.

A primary evoked compound action potential (ECAP)-guided closed-loop stimulation program was configured for routine daily use, primarily for the chronic treatment of neuropathic pain. This program served as the patient’s standard background stimulation throughout the day. During subsequent outpatient follow-up, additional task-specific stimulation programs were developed to support functionally relevant motor activities, particularly sit-to-stand transitions, upright posture, and walking-related training. These programs were introduced after hospital discharge and were used selectively during physiotherapy and mobility-related activities rather than as continuous background stimulation.

**Figure 1:**
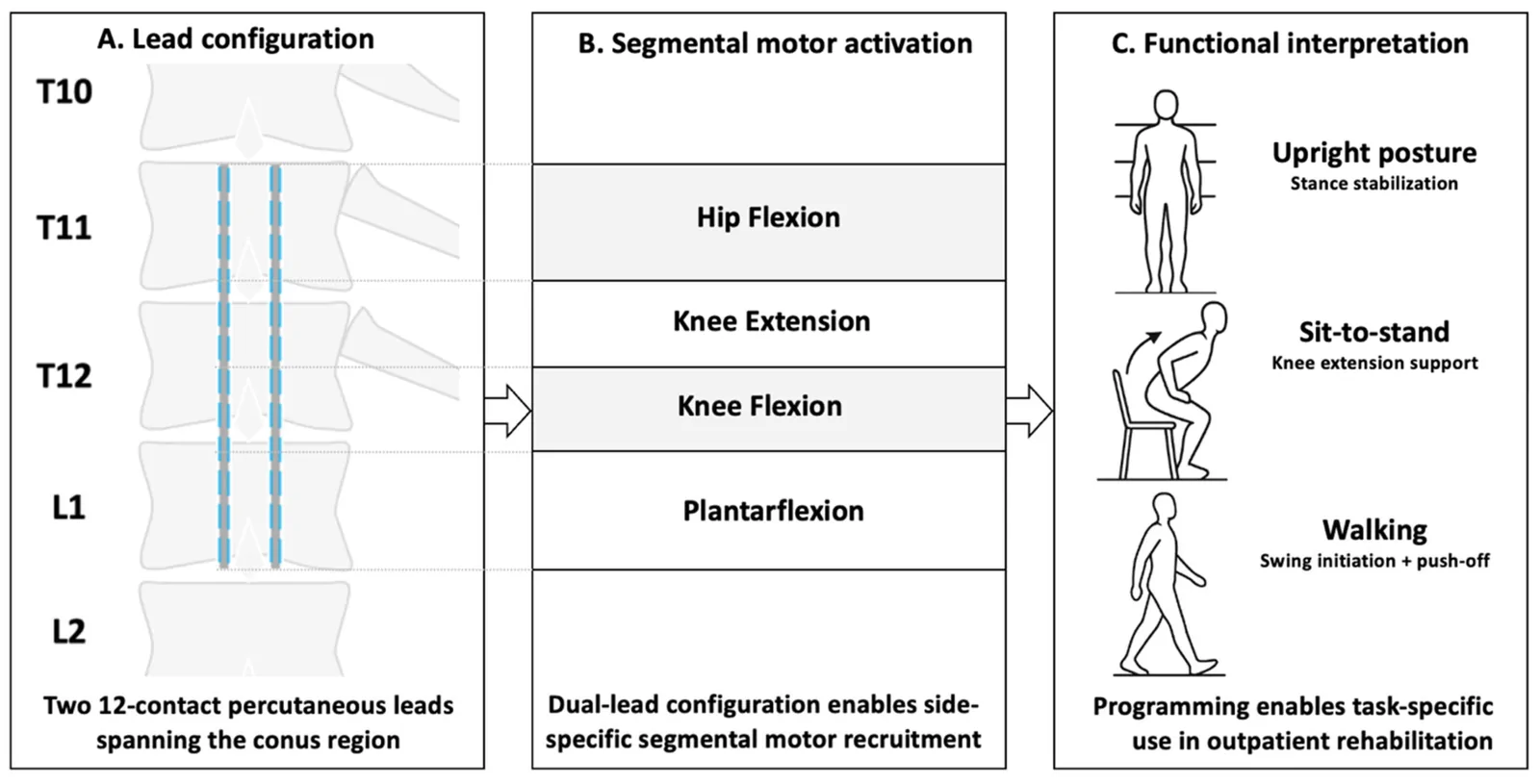
Segment-specific motor recruitment during postoperative stimulation mapping. During routine postoperative programming, stimulation of different electrode regions elicited reproducible motor responses corresponding to lumbosacral segmental organization. More proximal contacts predominantly facilitated hip flexion, mid-array contacts knee extension, and more distal contacts hamstring and plantarflexor activation. The two-lead configuration further enabled side-specific modulation of motor responses and informed the development of task-specific stimulation programs for standing and gait-related training.

Postoperatively, systematic stimulation mapping was performed during routine programming sessions. Different cathode–anode contact configurations were evaluated while varying stimulation parameters, including amplitude, pulse width, and frequency. Patient-reported paresthesia coverage and stimulation-evoked motor responses were assessed to characterize the functional effects of stimulation across the conus region. Stimulation of different electrode regions elicited reproducible, segmentally organized motor activation patterns, with more proximal contacts predominantly inducing hip flexion, mid-array contacts facilitating knee extension, and more distal contacts eliciting hamstring and plantarflexor activation (Figure 1). These observations were used to generate a functional map of the stimulated region, refine stimulation settings, and develop individualized task-specific programs for upright posture, sit-to-stand transitions, and walking-related training.

The two parasagittal percutaneous epidural leads also allowed side-specific modulation, with different electrode configurations producing preferential motor responses in the left or right lower extremity. This enabled more targeted, functionally tailored programming during rehabilitation.

Programming adjustments were made iteratively during routine follow-up visits based on the patient’s clinical needs and responses to stimulation rather than according to a predefined protocol. The distinction between a continuously used analgesia-focused program and intermittently applied task-specific motor programs reflects the clinically pragmatic approach used to integrate neuromodulation into outpatient care. Representative stimulation settings used for the analgesia-focused and task-specific programs are summarized in Table A1.

## 5. Rehabilitation Pathway

Following discharge, all postoperative care and rehabilitation were conducted in an outpatient setting, without a structured postoperative inpatient rehabilitation program. Physiotherapy was continued with the same outpatient physiotherapist who had treated the patient prior to implantation, with two 1.5 h sessions per week. This represented an increase from approximately 1.5 h of physiotherapy before implantation to 3 h per week after implantation.

From the first postoperative physiotherapy session until the six-month follow-up, the patient completed approximately 72 h of physiotherapy. No specific exercise regimen was mandated as part of this report; rather, physiotherapy followed the patient’s established outpatient rehabilitation routine and included mobility, strengthening, and functional training.

Task-specific stimulation programs were incorporated into therapy sessions and mobility-related activities as tolerated (see Table A1). Potential adjustments to stimulation settings were identified based on patient feedback and observations by the treating physiotherapist. When clinically indicated, adjustments were implemented during routine follow-up visits or, when necessary, at the rehabilitation facility by device representatives following prior approval and specification by the treating clinical team. This approach reflected routine outpatient care rather than a protocol-driven rehabilitation intervention.

## 6. Outcome Measures

Lower-extremity muscle strength was assessed using standardized handheld dynamometry (DynaMo, VALD, Brisbane, Australia). Handheld dynamometry provides a quantitative measure of isometric muscle strength by recording the maximal force generated against examiner-applied resistance. Predefined muscle groups including hip flexion, hip extension, hip abduction, knee flexion, and knee extension were tested bilaterally according to a standardized protocol with consistent patient positioning, limb alignment, and examiner-applied resistance across all visits. All measurements were performed by the same examiner to minimize inter-rater variability and were conducted before physiotherapy or other physically demanding assessments on the respective study day.

Assessments were conducted before implantation and at monthly intervals with the outpatient physiotherapist. At each postoperative assessment, measurements were obtained under both stimulation-OFF and stimulation-ON conditions, with ON measurements performed using the respective functional stimulation program rather than the continuous pain-focused program. This allowed within-session assessment of stimulation-associated changes in force generation. Across follow-up, 128 individual dynamometry measurements were obtained, with isometric force recorded in Newtons (N) for each tested muscle group. Given the reduced sensitivity of handheld dynamometry at low force levels, measurements in this range were interpreted cautiously and are presented descriptively.

Complementary clinical outcome measures assessed mobility, neurological function, quality of life, bowel and bladder symptoms, and pain. Patient-reported outcome measures included the Neurogenic Bowel Dysfunction (NBD) score, Qualiveen questionnaire, and the World Health Organization Quality of Life-BREF (WHOQOL-BREF) questionnaire [10,11,12,13]. Additional clinical assessments included the 10 m walk test (10MWT) and the International Standards for Neurological Classification of Spinal Cord Injury (ISNCSCI), from which the Lower Extremity Motor Score (LEMS) was derived. No dedicated neuropsychological, cognitive, or psychiatric assessment was performed. This case report was prepared in accordance with the CARE reporting guidelines [14].

## 7. Results

Following the trial stimulation phase, the patient reported clinically meaningful pain relief, with NRS scores decreasing from 5 to 8/10 preoperatively to 3/10 during the trial period. Pain reduction remained stable throughout follow-up. Stimulation was well tolerated during routine daily use and during physiotherapy- and mobility-related activities. The stimulation programs were integrated into routine outpatient physiotherapy without relevant practical limitations or intolerance reported by the patient or physiotherapy team.

**Figure 2:**
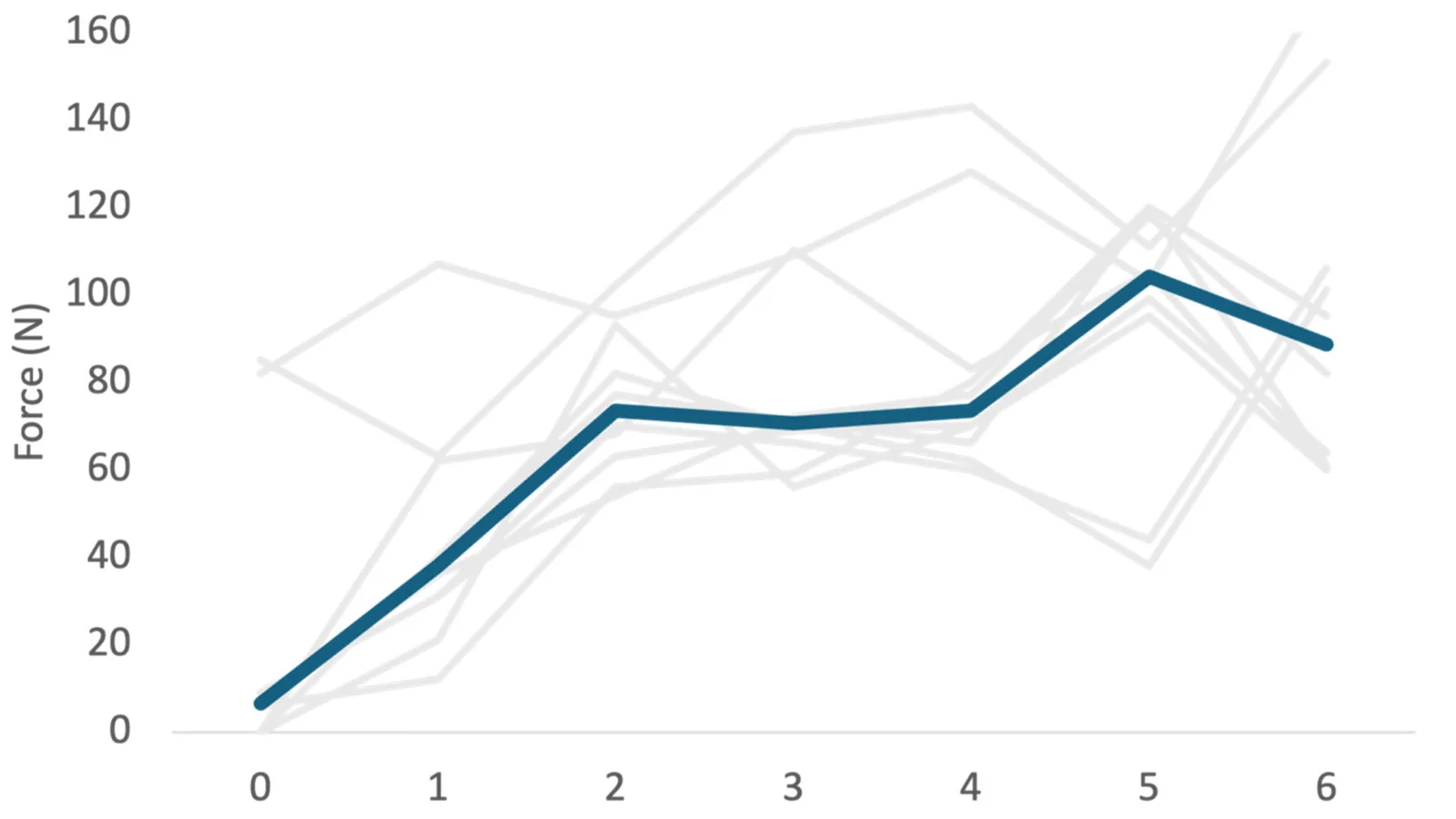
Longitudinal change in stimulation-OFF lower extremity muscle strength. Handheld dynamometry measurements obtained with stimulation OFF are shown across the six-month follow-up. Each thin line represents an individual left- or right-sided muscle measurement from hip flexion, hip extension, hip abduction, knee flexion, or knee extension. The thick line indicates the median across all measurements at each time point. Absolute force values are shown; month 0 represents the pre-implantation baseline.

**Figure A1:**
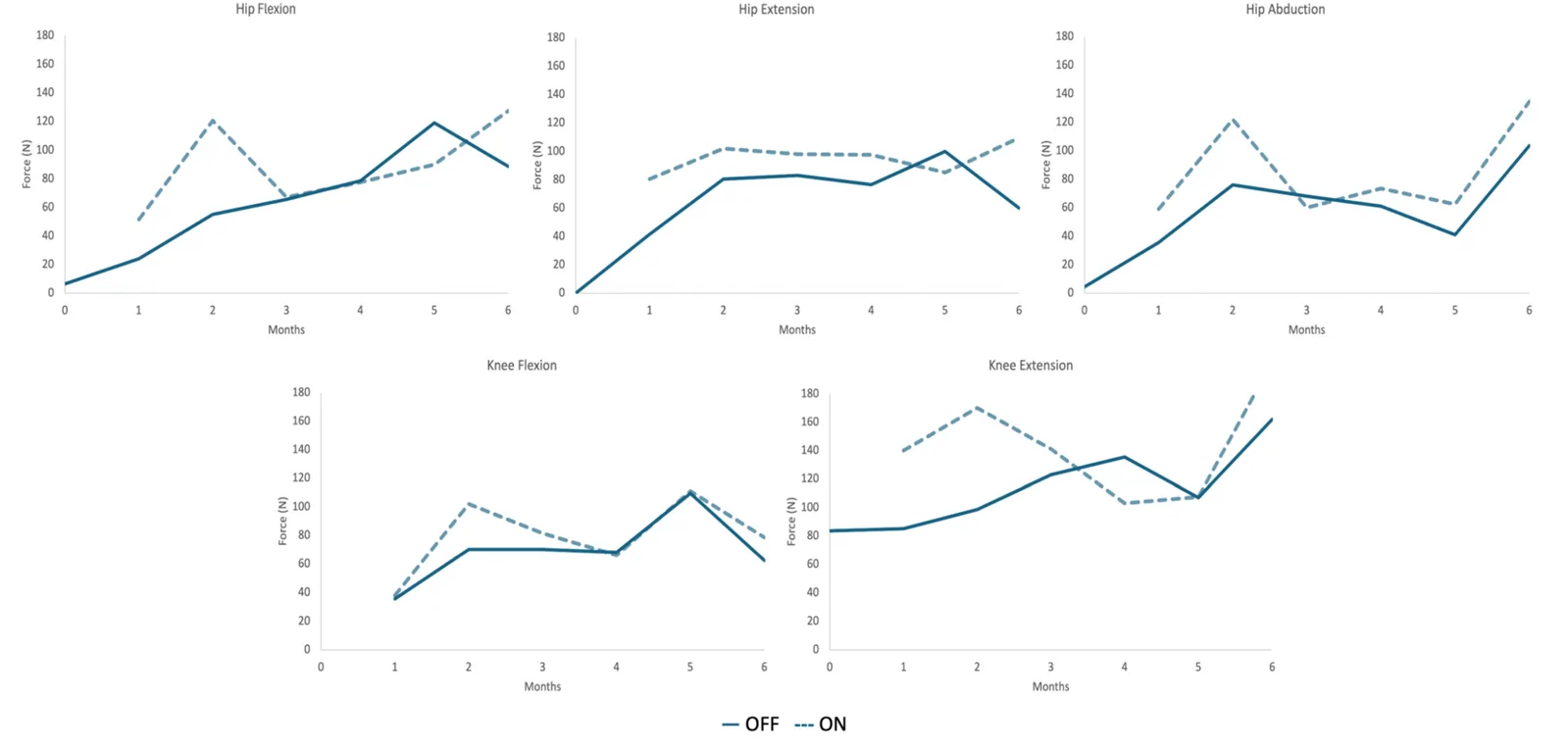
Individual muscle trajectories. Longitudinal handheld dynamometry measurements for individual lower-extremity muscle groups are shown as bilateral averages. Solid lines represent stimulation-OFF and dashed lines stimulation-ON conditions. Axes are standardized across panels to facilitate comparison between muscle groups. Substantial variability between muscle groups and across assessment sessions was observed. Stimulation-ON measurements were not obtained at baseline (pre-implantation), and baseline measurements for knee flexion were not recorded.

Serial handheld dynamometry was analyzed in two complementary ways to distinguish longitudinal changes in motor performance from acute stimulation-associated effects. First, force measured with stimulation-OFF was followed over six months and compared with the pre-implantation baseline (Figure 2). OFF-state force increased across the majority of individual muscle measurements and remained above baseline throughout follow-up. Although the magnitude and time course of improvement varied between muscle groups, OFF-state force increased over the first five months and remained above baseline. Individual ON- and OFF-state trajectories for each tested muscle group are provided in Figure A1.

**Figure 3:**
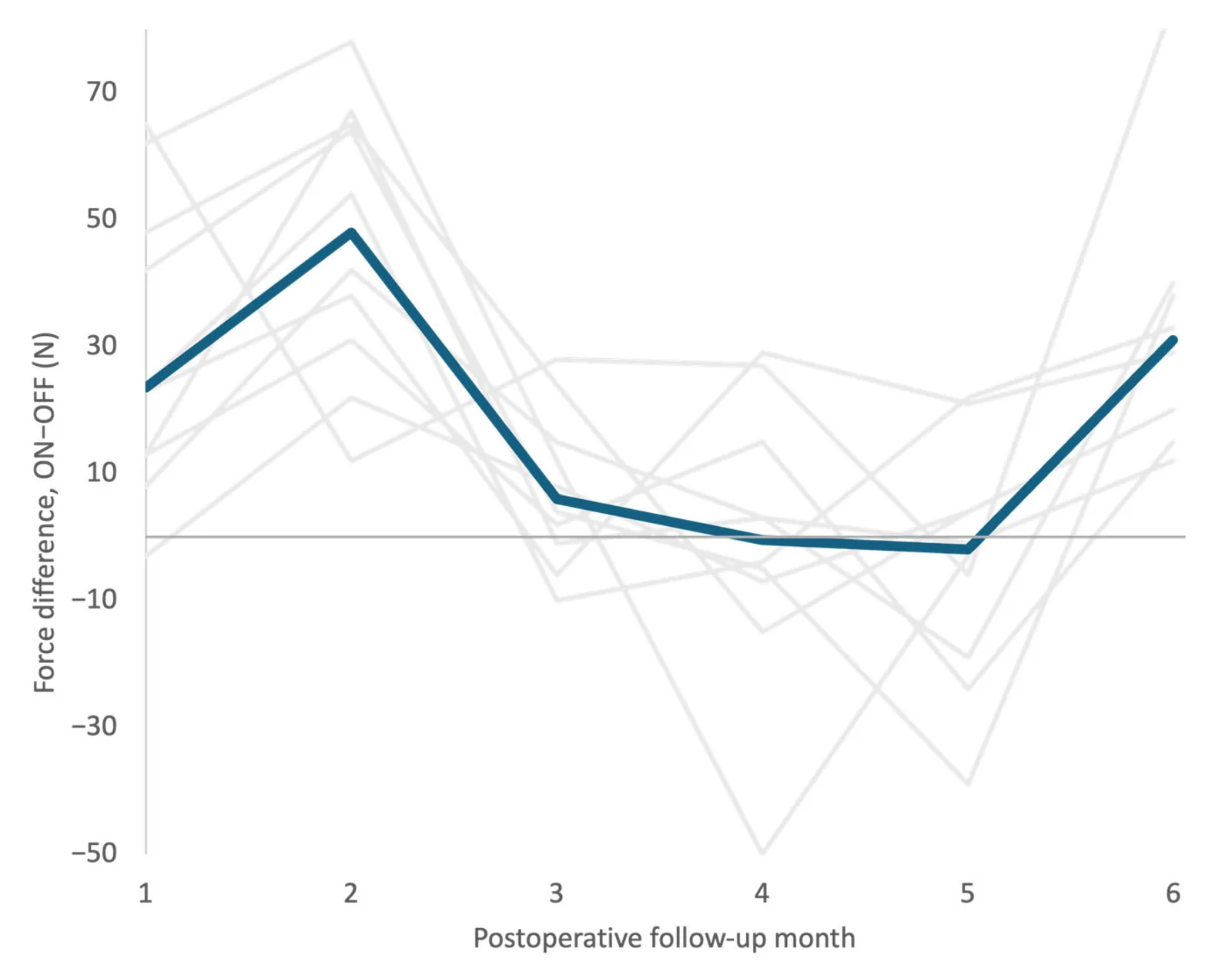
Within-session difference in lower extremity muscle strength between stimulation-ON and stimulation-OFF conditions across follow-up. At each postoperative assessment, lower-extremity muscle force was measured with the functional stimulation program ON and OFF, and the difference was calculated as ON minus OFF. Each thin line represents the ON–OFF difference for an individual left- or right-sided muscle measurement from hip flexion, hip extension, hip abduction, knee flexion, or knee extension. The thick line indicates the median ON–OFF difference across all measurements at each time point. Positive values indicate greater force with stimulation-ON, values around zero indicate little difference between conditions, and negative values indicate greater force during the OFF measurement. Data are pooled across muscle groups to illustrate the overall temporal pattern of within-session ON–OFF differences; individual muscle-specific ON and OFF trajectories are shown in Figure A1.

Second, stimulation-associated effects were assessed at each postoperative visit by comparing force measured within the same session with the functional stimulation program ON versus OFF (Figure 3). Positive ON–OFF values indicate greater force with stimulation ON, values around zero indicate little difference between conditions, and negative values indicate greater force during the OFF measurement. During the first two months, force was greater with stimulation ON than OFF across most tested muscle groups.

During intermediate follow-up, within-session ON–OFF differences diminished, approaching zero or becoming negative in several muscle groups, while absolute force measured with stimulation OFF continued to increase (Figure 2). Thus, the decreasing ON–OFF difference occurred in the context of improving OFF-state strength rather than a return toward pre-implantation motor performance. At month 6, positive ON–OFF differences became more apparent again, coinciding with a decline in OFF-state force in several muscle groups while ON-state force was largely maintained. The two analyses therefore describe complementary aspects of motor performance: Figure 2 shows longitudinal changes in force measured without active stimulation, whereas Figure 3 shows within-session differences between stimulation-ON and stimulation-OFF conditions at each postoperative assessment. Given the exploratory single-patient design and reduced precision of handheld dynamometry at low force levels, these findings are presented descriptively.

Complementary functional and patient-reported outcomes also changed over the six-month follow-up. Lower Extremity Motor Score (LEMS) increased from 36 to 38 points over six months, while the 10 m walk test decreased from 69 to 57 s. Despite remaining primarily wheelchair-dependent for everyday mobility, the patient demonstrated measurable improvements in voluntary lower-extremity motor function and assisted ambulation. Activities of daily living were not systematically assessed using a dedicated instrument, and therefore no conclusions regarding changes in overall independence can be drawn. Pain intensity decreased from 5 to 8/10 preoperatively to 3/10 at follow-up, accompanied by improvements in bowel function (NBD from 6 to 1), urinary-related quality of life (Qualiveen from 1.03 to 0.875), and physical quality of life (WHOQOL-BREF physical domain from 36 to 50) (Table A2). The improvement in NBD score reflected improved bowel movement frequency, resolution of constipation symptoms, and reduced lifestyle impact of bowel dysfunction. Improvements in Qualiveen were observed in items related to travel restrictions, planning of activities around urinary problems, and lifestyle adaptations due to urinary dysfunction. The improvement in physical quality of life was accompanied by reduced interference of physical pain with activities, a reduced perceived need for medical treatment to function in daily life, and improved sleep. Psychological well-being, assessed using the WHOQOL-BREF Psychological Health domain, also improved from 63 at baseline to 71 at six months, with the largest gains relating to greater satisfaction with self and improved acceptance of bodily appearance.

## 8. Discussion

This report describes the integration of percutaneous SCS of the conus region, implanted for an established pain indication, into an outpatient rehabilitation pathway in a patient with chronic incomplete SCI. Within this real-world setting, repeated outpatient assessments captured stimulation-associated differences and longitudinal changes in motor performance, along with changes in functional and patient-reported outcomes.

One of the main observations of this report was the combination of early stimulation-associated increases in force generation and longitudinal increases in OFF-state strength relative to baseline. During early follow-up, stimulation-ON measurements showed higher force output than stimulation-OFF measurements across most tested muscle groups. During intermediate follow-up, ON–OFF differences diminished substantially and, in some cases, became negative, while absolute OFF-state strength increased across multiple muscle groups. Longitudinal increases in force were therefore also observed during stimulation-OFF assessments. At month six, ON–OFF differences became more apparent again; however, this coincided with a decline in OFF-state force in several muscle groups while ON-state force was largely maintained. The increased ON–OFF difference at this time point therefore appears to reflect, at least in part, lower OFF-state performance rather than a renewed increase in stimulation-associated force generation. Overall, these findings suggest that acute stimulation-associated facilitation may coexist with longitudinal improvements in OFF-state motor performance. Potential contributors include activity-dependent adaptation and training-related plasticity, although these mechanisms cannot be established from the present case. Proposed mechanisms of SCS-associated motor facilitation include increased excitability of spinal interneuronal networks, facilitation of residual descending pathways, and activity-dependent plasticity associated with repeated motor training [15,16,17,18]. In addition, increased physical activity during the follow-up period may have contributed to peripheral adaptations, including improved muscle conditioning or increases in muscle mass, although these parameters were not formally assessed.

Previous studies have demonstrated that SCS can facilitate voluntary movement and functional recovery after SCI, primarily in highly controlled experimental environments or intensive inpatient rehabilitation settings [5,6,8]. Many of these approaches employ spatiotemporal stimulation paradigms designed to deliver phase-specific activation patterns synchronized with gait cycles, often relying on custom research platforms with external sensor integration and real-time stimulation control that are not routinely available in clinical practice. In contrast, the present report documents stimulation-associated motor differences and longitudinal motor changes using clinically available stimulation programs without real-time modulation and within routine outpatient care. Although such simplified approaches do not provide the same degree of task-specific coordination as spatiotemporal stimulation paradigms, this observation warrants further investigation of clinically available neuromodulation strategies outside specialized research settings.

The segmentally organized motor responses observed during programming are anatomically consistent with stimulation of the conus region. In contrast to experimental motor restoration approaches, which may rely on advanced imaging techniques for visualization of individual nerve roots, intraoperative electrophysiological mapping, or highly specific stimulation paradigms, lead placement in this case was based on standard preoperative MRI for anatomical localization combined with paresthesia-guided intraoperative positioning. Contact-dependent activation of hip, knee, and distal muscle groups provided a functional map that was used to develop task-specific stimulation programs. The use of two percutaneous leads additionally allowed side-specific modulation of motor responses during rehabilitation.

From a clinical perspective, the novelty of this case lies in the feasible integration of motor-oriented mapping and programming into clinically indicated percutaneous SCS using a commercially available system and routine outpatient rehabilitation, rather than a specialized experimental rehabilitation environment. Stimulation was well tolerated during daily use and physiotherapy, and no major barriers to integration into routine ambulatory care were encountered. No stimulation-associated adverse effects were observed during follow-up. Although scalability and effectiveness across a broader SCI population cannot be inferred from a single case, the pragmatic nature of this approach supports its prospective evaluation in larger cohorts.

Management of chronic pain after SCI is multimodal and includes pharmacological, rehabilitative, and psychological approaches. Pharmacological options such as pregabalin, gabapentin and amitriptyline have demonstrated efficacy for SCI-associated pain, although treatment response and tolerability vary considerably between individuals [19,20]. Non-pharmacological and psychological interventions may provide additional benefit as part of a broader pain management strategy, although the evidence for some of these approaches remains limited [21,22]. SCS should therefore be considered within a multimodal treatment framework rather than as an isolated therapeutic approach. For patients with chronic incomplete SCI who are candidates for SCS because of refractory neuropathic pain, motor-relevant effects may represent an additional domain worth systematically assessing. However, this single case does not support SCS implantation solely for motor restoration, but rather provides a rationale for prospective evaluation of such effects in appropriately selected patients.

Several limitations must be acknowledged in this report. This is a single-patient observation without a control condition, and the findings cannot be generalized to other patients with SCI or used to establish a causal relationship between SCS and the observed motor changes. Improvements may have been influenced by concurrent rehabilitation, placebo effects, or natural variability. Although dynamometric assessments were performed by the same examiner using a standardized protocol, test–retest variability across different assessment days was not formally quantified, and day-to-day fluctuations in motor performance cannot be excluded. Physiotherapy exposure increased during follow-up, which may have contributed to the observed improvements. Similarly, the sustained reduction in neuropathic pain may have contributed to improved overall functional performance. However, serial within-session dynamometry under stimulation-ON and stimulation-OFF conditions allowed acute stimulation-associated differences in force generation to be assessed independently of between-day variability. Programming was individualized and not standardized, and outcome assessments were not blinded, limiting causal attribution and introducing the possibility of observer bias. While the within-session ON–OFF assessments provide a within-patient comparison of acute stimulation-associated effects, they do not substitute for a blinded controlled design. Handheld dynamometry has reduced precision at low force levels, particularly in weaker muscle groups. Although the patient remained primarily wheelchair-dependent for community mobility at six months, objective functional measures demonstrated meaningful gains, including faster assisted walking performance, a modest improvement in LEMS, and improvements in bowel function, pain, and physical quality of life. These findings suggest clinically relevant functional gains despite the absence of independent ambulation. In addition, follow-up was limited to six months. Although longitudinal improvements were repeatedly observed under stimulation-OFF conditions during this period, their durability beyond six months and following prolonged withdrawal of stimulation remains unknown.

## 9. Conclusions

This case illustrates how clinically indicated percutaneous SCS can be combined with motor-oriented programming within routine outpatient rehabilitation. The observed stimulation-associated motor effects and longitudinal changes in stimulation-OFF strength are limited to this individual patient and cannot establish efficacy or causality. Prospective controlled studies are needed to determine the reproducibility and clinical relevance of these observations in chronic incomplete SCI.

## Data Availability

The data supporting the findings of this case report are available from the corresponding author upon reasonable request. The data are not publicly available due to patient privacy and ethical considerations.

## Appendix A. Additional Clinical and Stimulation Data

**Table A1:** Stimulation programs and representative settings used during outpatient follow-up.

Program	Clinical Purpose	Active Contacts	Frequency (Hz)	Pulse Width (µs)	Amplitude (mA)	Control Mode	Typical Use
1	Neuropathic pain control	2+, 3−, 4+	40	260	1	ECAP-guided closed-loop	Continuous daily background stimulation
2	Transfer/sit-to-stand support	13+, 16−, 19+	40	390	1.2	Open-loop	Used selectively during transfer tasks and sit-to-stand training
3	Upright posture/stance stabilization	1−, 2−, 3−, 4− 8+, 9+, 10+, 11+, 12+ 13−, 14−, 15−, 16− 21+, 22+, 23+, 24+	50	450	0.7–1.5	Open-loop	Used selectively during standing and postural exercises
4	Gait-related training	4−, 6−, 8+ 13+, 14−, 15+	20	350	1.1–1.7	Open-loop	Used selectively during gait-related training

Programming parameters were individualized and iteratively adjusted during follow-up according to patient-reported effects, observed motor responses, and functional training goals. Values shown represent the stimulation ranges and representative contact configurations used during the follow-up period rather than fixed protocol-defined settings. Contacts 1–12 correspond to the right lead and contacts 13–24 to the left lead, numbered in cranio-caudal orientation.

**Table A2:** Complementary clinical and patient-reported outcomes at baseline and six-month follow-up.

Outcome Measure	Domain	Score Range/Units	Interpretation	Baseline	Month 6
ASIA Impairment Scale (AIS)	Neurological impairment	A–E	A = complete injury; B–D = incomplete injury; E = normal	D	D
Lower Extremity Motor Score (LEMS)	Voluntary lower extremity motor function	0–50	Higher values indicate better motor function	36	38
ISNCSCI sensory examination	Light touch/pinprick sensation	0–224 (combined)	Higher values indicate better sensory function	224	224
10 m-walk-test (10MWT)	Walking speed/mobility	Seconds	Lower values indicate faster walking performance	69	57
Qualiveen questionnaire	Urinary-related quality of life	0–4	Lower values indicate less bladder-related impact on quality of life	1.03	0.875
Neurogenic Bowel Dysfunction (NBD) score	Bowel function	0–47	Lower values indicate less severe bowel dysfunction	6	1
WHOQOL-BREF (physical health)	Quality of life	0–100	Higher values indicate better physical quality of life	36	50
WHOQOL-BREF (psychological health)	Quality of life	0–100	Higher values indicate better psychological quality of life	63	71
Numeric Rating Scale (NRS)	Pain intensity	0–10	Lower values indicate less pain	5–8	3

Outcome measures are presented descriptively because of the exploratory single-patient nature of this report. For multidomain questionnaires, the most clinically relevant domain(s) are shown.
